# Histone deacetylase inhibitor-temozolomide co-treatment inhibits melanoma growth through suppression of Chemokine (C-C motif) ligand 2-driven signals

**DOI:** 10.18632/oncotarget.2065

**Published:** 2014-06-06

**Authors:** Laura Gatti, Alexandra Sevko, Michelandrea De Cesare, Noemi Arrighetti, Giacomo Manenti, Emilio Ciusani, Paolo Verderio, Chiara M. Ciniselli, Denis Cominetti, Nives Carenini, Elisabetta Corna, Nadia Zaffaroni, Monica Rodolfo, Licia Rivoltini, Viktor Umansky, Paola Perego

**Affiliations:** ^1^ Molecular Pharmacology Unit, Fondazione IRCCS Istituto Nazionale dei Tumori, Milan, Italy; ^2^ Skin Cancer Unit, German Cancer Research Center (DKFZ), Heidelberg and Department of Dermatology, Venereology and Allergology, University Medical Center Mannheim, Ruprecht-Karl University of Heidelberg, Mannheim, Heidelberg, Germany; ^3^ Genetic Epidemiology and Pharmacogenomics Unit, Fondazione IRCCS Istituto Nazionale dei Tumori, Milan, Italy; ^4^ Laboratory of Clinical Pathology and Medical Genetics, Fondazione IRCCS Istituto Neurologico C. Besta, Milan, Italy; ^5^ Medical Statistics, Biometry and Bioinformatics Unit, Fondazione IRCCS Istituto Nazionale dei Tumori, Milan, Italy; ^6^ Immunotherapy Unit, Fondazione IRCCS Istituto Nazionale dei Tumori, Milan, Italy

**Keywords:** melanoma, CCL2, JNK, histone deacetlylase inhibition, temozolomide

## Abstract

Target-specific agents used in melanoma are not curative, and chemokines are being implicated in drug-resistance to target-specific agents. Thus, the use of conventional agents in rationale combinations may result in optimization of therapy. Because histone deacetylases participate in tumor development and progression, the combination of the pan-inhibitor SAHA and temozolomide might provide a therapeutic advantage. Here, we show synergism between the two drugs in mutant BRAF cell lines, in association with decreased phosphorylation of cell survival proteins (e.g., C-Jun-N-terminal-kinase, JNK). In the spontaneous *ret* transgenic mouse melanoma model, combination therapy produced a significant disease onset delay and down-regulation of Chemokine (C-C motif) ligand 2 (CCL2), JNK, and of Myeloid-derived suppressor cell recruitment. Co-incubation with a CCL2-blocking-antibody enhanced *in vitro* cell sensitivity to temozolomide. Conversely, recombinant CCL2 activated JNK in human tumor melanoma cells. In keeping with these results, the combination of a JNK-inhibitor with temozolomide was synergistic. By showing that down-regulation of CCL2-driven signals by SAHA and temozolomide *via* JNK contributes to reduce melanoma growth, we provide a rationale for the therapeutic advantage of the drug combination. This combination strategy may be effective because of interference both with tumor cell and tumor microenvironment.

## INTRODUCTION

Melanoma is an aggressive disease exhibiting a metastatic behavior and intrinsic resistance to treatment, features leading to poor prognosis. In spite of the development of target-specific agents (i.e., BRAF inhibitors) which have improved progression-free survival and responsiveness to immune intervention (e.g., anti CTLA4/PD1 antibodies, Ipilumab/Yervoy), metastatic melanoma is still incurable [1-3]. Resistance to therapy is due to multiple factors which include both tumor cell alterations during tumor development and progression, and extrinsic factors present in the microenvironment, including chemokines such as Chemokine (C-C motif) ligand 2 (CCL2)/monocyte chemotactic protein-1 [4, 5]. Deregulation of the balance between melanocyte stem cell proliferation and differentiation into melanocytes, a process implicating multiple genes and epigenetic factors, is thought to result in melanoma initiation and progression [6].

In melanoma, deregulation of survival pathways as a consequence of BRAF mutation is a frequent event [7]. Because BRAF signals through the MAPK pathway, such a pathway is recognized as relevant in melanoma cell survival. Besides being activated by BRAF mutation, the MAPK pathway hyper-activation can be achieved by de-regulated expression of tyrosine kinases including those of the HER family and RET [8, 9]. Moreover, besides MAPK activation, gene-specific mutation signals triggered by mutant BRAF on other pathways have been reported to lead to transcriptional changes [10]. Altered DNA methylation or acetylation may participate to regulate such changes [11].

Malignant transformation is also driven by epigenetic mechanisms that involve histone deacetylases (HDAC) which remove acetyl groups from histones, thereby promoting chromatin condensation and acting as transcription repressors [12]. Conversely, acetylation of histones promotes a relaxed chromatin structure, allowing transcriptional activation. Histone modifications regulate gene-expression patterns underlying a variety of biological processes including response to stress and to DNA damage [12]. In addition, HDAC have non-histone substrates, including regulators of transcription, heat shock protein-90, and α-tubulin [13]. Since HDAC may be implicated in the silencing of growth regulatory pathways relevant to sustain tumor progression and drug resistance [14], HDAC inhibitors may improve response to antitumor agents.

The only cytotoxic agent approved by the US Food and Drug Administration for the treatment of melanoma is the triazine dacarbazine [15]. Temozolomide (TMZ) [16], an imidazotetrazine derivative of dacarbazine is an attractive agent for patients with unresectable metastatic malignant melanoma due to its oral route of administration and its ability to cross the blood brain barrier [17]; TMZ is currently undergoing clinical evaluation in melanoma (http://www.clinicaltrials.gov). Novel therapeutic options are needed to improve the activity of such clinically available agents and to define alternative treatments for patients recurring after BRAF inhibitors (i.e., bearing mutant BRAF tumors) as well as for patients bearing tumors with wild-type BRAF. In this regard, HDAC inhibitors represent promising therapeutic agents because they are expected to act on tumor cells and their microenvironment as they are endowed with immunomodulatory properties [18]. Indeed, it has been shown that HDAC inhibitors reduce tumor and immune cell ability to produce various inflammatory factors [19-22]. It is known that persistent production of inflammatory factors under chronic inflammatory conditions in the tumor microenvironment strongly supports the generation, recruitment and activation of myeloid-derived suppressor cells (MDSC) [23-25]. Many anti-inflammatory effects of histone deacetylase inhibitors are mediated through effects on antigen-presenting cells, including monocytes [26]. Suppression of granulocyte chemotactic protein-2 (GCP-2), monocyte chemotactic protein-2 (MCP-2) and macrophage migration inhibitory factor (MIF) has been reported in rheumatoid arthritic synovial fibroblastic cells [27]. Although the pan-HDAC inhibitor suberoylanilide hydroxamic acid (SAHA, vorinostat) has been approved for the treatment of cutaneous T lymphomas [28], its role in the treatment of melanoma is still under investigation (e.g., ClinicalTrials.gov Identifier: NCT01587352).

Based on this background, we hypothesize that SAHA could be useful in an attempt to improve the efficacy of the TMZ and we used a panel of human melanoma cell lines, expressing alterations found in tumors from patients resistant to BRAF inhibitors, to investigate the therapeutic potential of SAHA in combination with the clinically available TMZ. Given the immunomodulatory potential of SAHA, the combination was tested *in vivo* on the *ret* transgenic mouse model of spontaneous melanoma. Here, we describe the molecular correlates of the efficacy of the combination of SAHA and TMZ, and we propose that disruption of CCL2-driven signals by SAHA and TMZ may impair survival of human melanoma cells resulting in a synergistic drug interaction which in mice results in delayed disease onset.

## RESULTS

### The combination between temozolomide and the pan-HDAC inhibitor SAHA displays an improved effect in human melanoma mutant and wild-type BRAF cells

**Figure 1:**
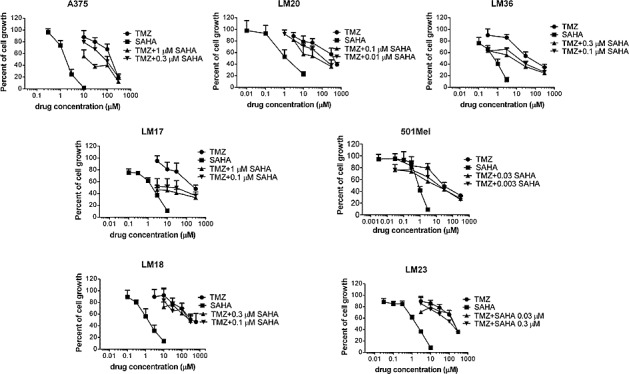
Cell sensitivity to temozolomide, SAHA or to their combination in human melanoma cell lines Cell sensitivity to temozolomide (TMZ), SAHA or to the combination was assessed by growth inhibition assays. Cells were exposed for 72 h to each drug alone or to the drug combination. At least three independent experiments are shown for each cell line.

A panel of human melanoma cell lines well characterized for their molecular features was used in this study. They included A375, LM17, LM20, LM36, 501Mel exhibiting the *BRAF^V600E^* mutation, and two BRAF wild-type cell lines, LM18 and LM23. The LM20 and 501Mel cell lines display intrinsic resistance to the BRAF inhibitor PLX4032. LM20 cells carry amplifications of *CDNN1* and *CTNNB1*, and 501Mel cells display high *MET* expression [29] (data not shown). Cell sensitivity to TMZ and to the pan-HDAC inhibitor SAHA was variable among the cell lines (Table 1). The effect of their combination was tested by the Chou and Talalay method in which a CI lower than 1 indicates synergism. Under such experimental conditions, a favourable drug interaction was observed in the different cell lines irrespectively of the relative level of sensitivity to TMZ or to SAHA (Figure 1). Indeed, a synergistic drug interaction was particularly evident in the five studied mutant BRAF cells – including established cell lines and cell lines recently derived from patients - as supported by the CI values (Supplementary Figure S1)

**Table 1:** Sensitivity of melanoma cell lines to temozolomide and SAHA^a^

Cell line	IC50 72 h (μM)	
	TMZ	SAHA
A375	152.8 ±19.7	1.77 ± 0.5
501Mel	44.97 ± 26.9	0.88 ± 0.3
LM17	248.39 ± 80.2	1.71 ± 0.5
LM20	405.6 ± 130.0	2.33 ± 1.4
LM36	74.0 ± 32.4	0.64 ± 0.2
LM18	296.9 ± 86.9	1.36 ± 0.5
LM23	163.3 ± 42.9	1.86 ± 0.8

a Cell sensitivity to drug was assessed by growth inhibition assay. Twenty-four hours after seeding, cells were exposed for 72 h to temozolomide (TMZ) or SAHA and were counted at the end of treatment. Mean ± SD of at least 3 independent experiments.

### Analysis of proteins involved in survival-pathways reveals consistent down-regulation of JNK activation upon combined treatment

**Figure 2:**
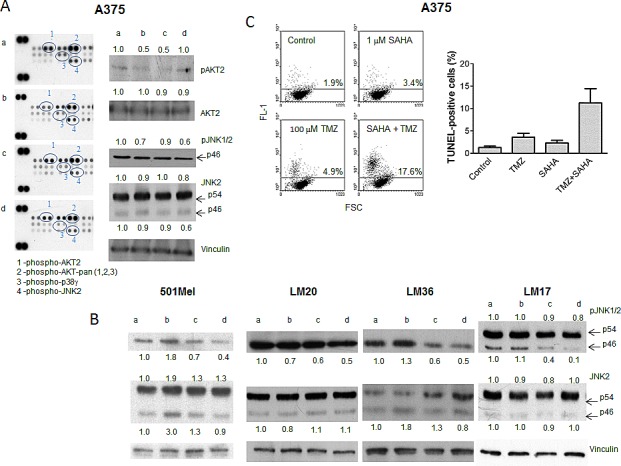
Tumor cell response to temozolomide, SAHA or their combination in human melanoma cell lines Cells were incubated with single drugs or their combination and processed 24 h later for Human Phospho MAPK Proteome Profiler (A), and Western blot analyses of phospho-JNK1/2 and JNK2 (B), or 72 h later for TUNEL assays (C). Small case letters refer to control (a), SAHA (b), temozolomide (TMZ) (c) or combination (d) treatments. 501Mel cells were exposed to 0.1 μM SAHA, 30 μM TMZ or to their combination (B). A375 cells were exposed to 1 μM SAHA, 30 μM TMZ, or to their combination (A, B); LM20 cells were exposed to 0.1 μM SAHA, 600 μM TMZ, or to their combination (B); LM36 cells were exposed to 0.1 μM SAHA, 30 μM TMZ, or to their combination (B); LM17 cells were exposed to 1 μM SAHA, 300 μM TMZ, or to their combination (B). Control loading is shown by vinculin. The ratio between the intensity of the indicated bands versus vinculin is reported (B). Quantitative analysis of apoptosis induction by TUNEL assays in A375 cells. Dot plots from a representative experiment and a graph from three independent experiments are shown; *P* = 0.032, unpaired t test of values from control versus combination-treated cells (C).

We hypothesized that the effect of the drug combination might be due to inhibition of the MAPK pathway resulting in growth inhibition (see Figure 1) and/or apoptosis. Thus, the A375 cell line was used to profile response to treatment with particular reference to the MAPK pathway (Figure 2A). We hybridized lysates of A375 cells treated with SAHA, TMZ or their combination with antibody arrays (Figure 2A, left panel). Exposure to SAHA produced a decrease in phospho-AKT levels, an effect that was more pronounced for the AKT2 isoform for which the down-regulation was still evident in cells treated with TMZ and with the drug combination. Exposure to the combination decreased the levels of phospho-JNK2 and phospho-p38γ. Western blot validation analysis evidenced a decrease of phospho-AKT2 (Figure 2A, right panel). The most frequent and consistent modulation was the down-regulation of phospho-JNK1/2 found in BRAF mutant cell lines upon the combined treatment (Figure 2A, right panel, Figure 2B, and data not shown). These data suggest a preferential impact of the combined treatment on JNK activation status.

The combination treatment resulted in an increase in apoptosis in A375 cells (Figure 2C) and in other cells lines (Supplementary Figure S2). Although in some models there was no evidence of increased apoptosis 72 h after drug exposure, apoptosis was observed 144 h after treatment (e.g., in LM36 cells), indicating that cell death could be a late event.

### Combination therapy produces a disease onset delay in the spontaneous *ret* transgenic mouse melanoma model associated with down-regulation of JNK activation in tumors

*Ret* transgenic mice which spontaneously develop melanoma were used. Because plasma LDH is considered a melanoma prognosis biomarker in humans, to characterize the model and to investigate the potential association between plasma LDH and disease in mice undergoing melanoma development, we measured LDH values in cases and control mice over time. Logistic regression analysis showed a borderline association between disease status and LDH values (data not shown). Supplementary Table S1 reports some descriptive statistics of the variable LDH in cases and controls. The box-plots (Supplementary Figure S3) describing the distribution of LDH in transgenic mice bearing melanoma (cases) and healthy mice (controls) show the increased LDH value observed in cases. Thus, this model showed some similarities with the human disease and was considered more useful than xenograft models because of the presence of a competent immune system.

**Figure 3:**
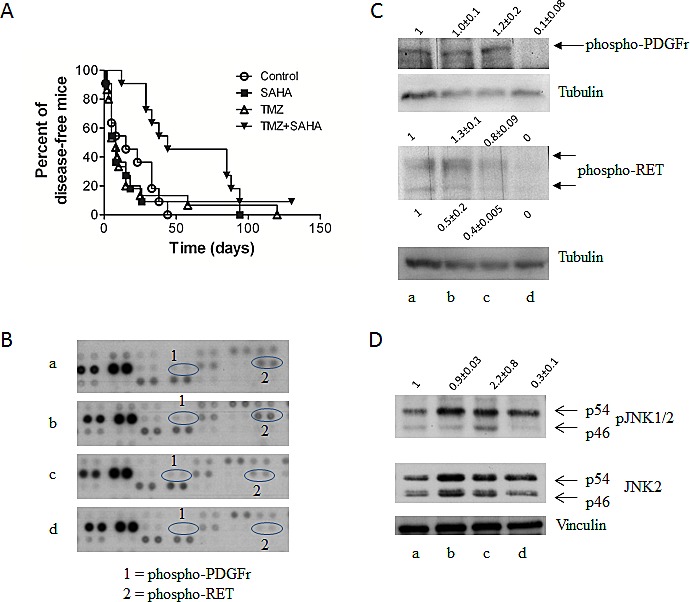
*In vivo* studies (A) Antitumor activity as shown by Kaplan Meier plots of the percentage of tumor-free mice over time. Mice were treated with temozolomide (TMZ) (50mg/kg qdx5) or SAHA (100mg/kg qdx5/wx4w) and their combination. Circles, control mice; squares, SAHA-treated mice; upright triangles, TMZ-treated mice, downright triangle, drug combination. Experimental groups consisted of 11-16 mice. (B) Phosphorylation of proteins involved in tumor cell survival as assessed by the mouse Phospho-RTK Proteome Profiler using lysates of tumors from control (a), SAHA (b), TMZ (c) or combination-treated (d) mice. Mice were treated as described above for 5 days and they were sacrificed 5 days later. Tumor cells were processed for total protein extraction. (C) Validation of Proteome Profiler by Western blotting. Analysis of phospho-PDGF receptor and phospho-RET in tumor specimens from control (a), SAHA (b), TMZ (c) or combination (d) treated mice. Control loading is shown by tubulin. Relative quantification mean values (± SD) of 3 independent experiments are shown. (D) Western blot analysis phospho-JNK1/2 and JNK2 in tumor specimens from control (a), TMZ (b), SAHA (c) or combination (d) treated mice. Control loading is shown by vinculin. Relative quantification values are shown.

When investigating the antitumor activity of the combination of SAHA and TMZ, mice bearing the *ret* transgene received SAHA, TMZ or both drugs (Figure 3A). Drug combination led to a significant delay in disease onset (*P* value of log-rank test: 0.0176). Mouse Phospho-RTK array analyses in tumors indicated a down-modulation of selected phospho-proteins after treatment (Figure 3B). Validation experiments confirmed down-regulation of phospho-PDGF receptor and phospho-RET levels (Figure 3C). Reduced phopho-JNK1/2 levels were observed upon combination treatment (Figure 3D), similarly to what observed in cell lines.

### Combination therapy produces down-regulation of the tumor chemokine CCL2 and of MDSC recruitment

**Figure 4:**
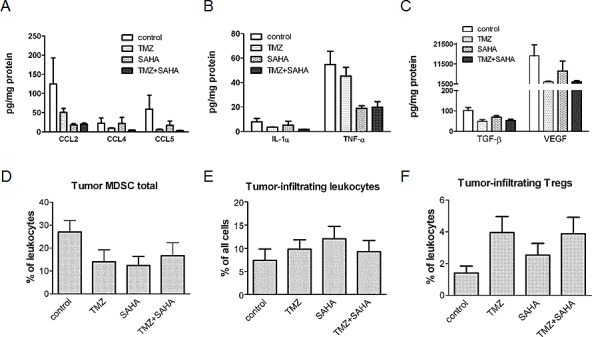
Effect of temozolomide, SAHA or their combination on the chronic inflammation tumor microenvironment-related factors Levels of chemo-attractant proteins (A), cytokines (B) and growth factors (C) after treatment with temozolomide (TMZ), SAHA or their combination were analyzed in tumor lysates (obtained from mice sacrificed 5 days after 5 day treatment) by multiplex technology except than for TGFβ1 which was measured using a single-plex kit. Results from the samples of 7-8 mice per group are presented as pg per mg of total proteins. Cumulative data from independent experiments for tumor-infiltrating MDSC (D), leukocytes, designated as CD45.2^+^ cells (E) and Tregs (F) measured by flow cytometry are expressed as the percentage within leukocytes (9-13 mice per group). Data are shown as mean ± SEM and were analyzed using the Kruskal-Wallis test. Kruskal-Wallis Bonferroni adjusted *P*-values: 0.04 (CCL2); 0.02 (CCL4); 0.02 (CCL5), 0.02 (IL1α), 0.08 (TNFα), 0.01 (TGFβ), 0.01 (VEGF).

To gain insights into the chronic inflammatory tumor microenvironment that could modulate tumor growth, we measured the levels of inflammatory factors in tumor lesions. The treatment with SAHA, TMZ or the combination was capable of modulating the pattern of tumor-associated chronic inflammatory mediators. Using the Kruskal-Wallis Test (KWT), we found a statistically significant association between the experimental groups and each of the considered continuous variable. A significant down-modulation was observed after exposure to the drug combination as compared to control animals for chemokines (CCL2, KWT Bonferroni adjusted *P*-values were: 0.04 for CCL2; 0.02 for CCL4; 0.02 for CCL5) (Figure 4A). Similarly, a significant decrease (KWT Bonferroni adjusted p-value: 0.02) was also found for IL-1α (Figure 4Β), which was shown to be a key mediator of sterile inflammation and being able to recruit MDSC towards the site of inflammation [23]. The concentration of another pro-inflammatory cytokine, TNF-α, showed a borderline significant down-modulation after combination treatment as compared to control group (KWT Bonferroni adjusted *P*-value: 0.08, Figure 4B). We found a significant reduction in the production of some immunosuppressive growth factors (KWT Bonferroni adjusted *P*-values were: 0.01 for TGF-β1; 0.01 for VEGF) in the drug combination treated groups as compared to controls (Figure 4C). Because HDAC inhibitors are expected to act on transcription regulation, the modulation at the protein level in part reflected that of mRNA in tumors, as shown by TaqMan arrays; down-modulation of CCL2 was confirmed also in human melanoma cells (Supplementary Figure S4).

All above mentioned factors mediate tumor-induced immune-suppression directly and *via* the generation, recruitment and activation of CD11b^+^Gr1^+^ MDSC [23]. Thus, the infiltration of immunosuppressive cells in the tumor microenvironment would also be diminished by treatment. Indeed, the population of CD11b^+^Gr1^+^ immature myeloid cells measured by flow cytometry tended to be reduced in the tumors upon all applied treatments as compared to the controls (Figure 4D) although no statistical significant results were found. Furthermore, the infiltration of these tumor samples with leukocytes (defined as CD45.2^+^ cells) remained similar in all considered groups (Figure 4E). A trend towards an increase of regulatory T cells was observed in tumors from single drug- and combination-treated mice (Figure 4F).

### The chemokine CCL2 can influence response to treatment *via* JNK

**Figure 5:**
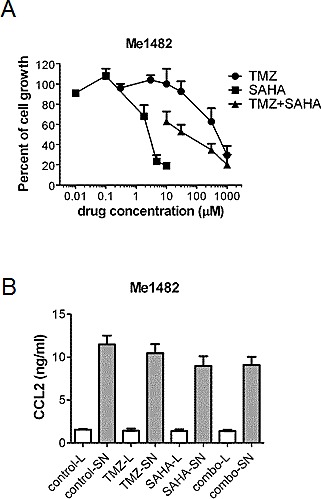
Effect of treatment with temozolomide, SAHA or their combination in murine melanoma cell line Me1482 (A) Modulation of cell growth of Me1482 cells by treatment. Cells were seeded and 24 h later they were incubated with temozolomide (TMZ), SAHA or their combination (with a subtoxic SAHA concentration of 1.8 μM) for 72 h. Cell sensitivity to drugs was assessed by cell growth inhibition assay. (B) ELISA of CCL2 levels in Me1482 cell lysates (L) and culture medium (SN) after 6 h exposure to the single drugs (30 μM TMZ; 1.8 μM SAHA) or to their combination.

**Figure 6:**
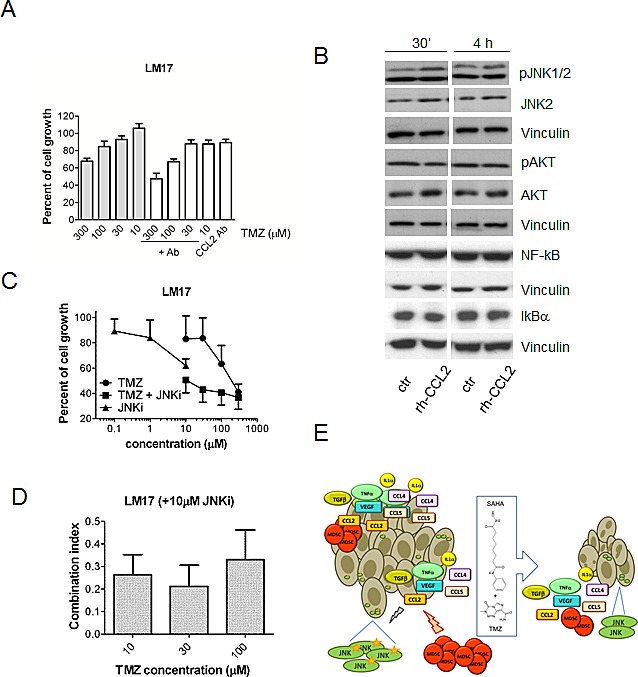
Role of CCL2 in response to treatment with temozolomide, SAHA or their combination in human melanoma cells (A) Effect of a 30 min pre-treatment with the anti-CCL2 antibody (5 μg/ml) on sensitivity of LM17 cells to temozolomide (TMZ, cell growth inhibition assays, 72 h exposure). Statistically significant difference (by Kruskal-Wallis test, KWT) was observed between antibody (Ab)/TMZ and TMZ alone at 100 μM (KWT p-value: 0.020) and 10 μM (KWT *P*-value: 0.04). (B) Western blot analysis of JNK, phospho-JNK, NF-kB, IKBα, AKT and phospho-AKT in LM17 cells exposed to 20 ng/ml recombinant CCL2 (rh-CCL2) for 30 min or 4 h. Control loading is shown by vinculin. For phospho-JNK upper band, relative quantification mean values (± SD) of 3 independent experiments are: 1 (control 30'), 2 ± 0.2 (rh-CCL2 30'), 1 (control 4 h), 1.7 ± 0.3 (rh-CCL2 4 h). (C) Sensitivity of LM17 cells to the combination of the JNK inhibitor SP600125 and TMZ (cell growth inhibition assays, 72 h exposure). (D) Drug interaction between the JNK inhibitor SP600125 (10 μM) and TMZ as shown by combination index (CI) values. The mean values (± SD) of 3 independent experiments are reported. (E) Overview of the mechanism of the improved effect of the combination of temozolomide and SAHA in melanoma. The chemokines and the immunesuppressive cells present at the tumor site are shown together with the effect of treatment. CCL2 is capable of activating survival pathways in melanoma cells and inhibition of melanoma growth by temozolomide (TMZ)-SAHA co-treatment is achieved though suppression of CCL2-driven signals. Beside CCL2 down-regulation, modulation of additional factors appears to contribute to the improved antitumor efficacy.

To better investigate the role of pro-survival signals in melanoma, we established a cell line (Me1482) from a tumor developed by mice (Supplementary Figure S5). When the effect of the combination of TMZ and SAHA was examined, a supra-additive effect was found according to the Chou and Talalay method (Figure 5A). Given the relevance of CCL2 in maintaining cell survival [24], we focused our attention on this chemokine. Control and drug-treated cells released CCL2 in the medium, suggesting that chemokines released by the tumor in the microenvironment can influence response to treatment (Figure 5B). Consistently, we observed a release of CCL2 in the medium of the human cells (141.9 ± 28.6 pg CCL2/μg proteins for A375 cells, 211.5 ± 17.9 pg CCL2/μg proteins for LM23 cells and 2.8 ± 1.4 pg CCL2/μg proteins for LM17 cells). In murine cells, a trend toward decrease of CCL2 levels in the supernatant was found, and this tendency was evident also in supernatants from human LM17 cells treated for 24 h, in which the drug combination produced a 68% decrease of CCL2 levels, whereas decrease after single agent treatments were around 40% (42% upon 0.1 μM SAHA exposure, 43% upon 30 μM TMZ exposure). In LM17 cells, pre-incubation with an anti-CCL2 antibody followed by TMZ exposure enhanced sensitivity to TMZ (Figure 6A). We hypothesized that CCL2 activated JNK, whose phosphorylation was decreased by the combination *in vivo* and *in vitro*. We observed that recombinant-CCL2 triggered JNK-activation in LM17 cells (Figure 6B). We tested whether a synergistic interaction could occur when combining TMZ and the JNK-inhibitor SP600125 and a marked synergistic interaction was observed as supported by CI values (Figure 6C-D).

## DISCUSSION

Based on the need for rationally improving the activity of clinically available agents, in the present study we used various molecularly characterized human melanoma cells, including cell lines with intrinsic resistance to the BRAF inhibitor vemurafenib, and *ret* transgenic mice to suppress tumor cell survival signals employing the pan-HDAC-inhibitor SAHA in combination with TMZ. The *ret* transgenic mouse model was chosen because it recapitulates the complexity of melanoma disease. In the used panel of melanoma cell lines, irrespectively of the inherent cell sensitivity to single agents, we found an improved effect of the drug combination as compared to single drugs, in association with the suppression of pro-survival signals. A feature of tumor cells exposed to the combination was the modulation of the phosphorylation profile of proteins involved in regulation of cell survival including AKT and members of the MAPK pathway. Of note, phosphorylation of JNK was reduced upon exposure to the combination in the cell lines in which the synergistic interaction occurred. Such an effect was associated with increased apoptosis in some cell lines. The synergistic interaction was more evident in cells characterized by BRAF mutation than in cell carrying wild-type BRAF, although the drug interaction on the latter cells was still favourable.

In addition, an improved response to the combination of SAHA and TMZ was evident in *ret* mice, which benefitted from combined treatment in terms of disease free-survival. The combination treatment led to a down-regulation of JNK activation in tumors, similarly to *in vitro* findings. When investigating the effect of the combination on the tumor inflammatory microenvironment, we found that levels of chemokines including CCL2 were significantly down-regulated in the primary tumors after the combined treatment compared to controls. Our data are reminiscent of what observed in a recent report in which down-regulation of CCL2 after treatment with the BRAF inhibitor PLX4720 was associated with reduced tumor growth [4]. Thus, the combination, while being therapeutically effective by improving disease free-survival could provide an approach to impair CCL2 production, an effect that may be exploited in the clinical setting to control melanoma growth.

The available evidence supports that CCL2, a chemotactic factor for monocytes and T lymphocytes plays key roles in tumorigenesis promotion in different tumor types [30, 31]. Intracellular JNK-signaling is implicated in CCL2 expression and CCL2-mediated proliferation [31, 32]. Consistently, cells lacking JNK1/2 were shown to display reduced levels of *ccl2* mRNA [33]. Because a decrease in the levels of CCL2 was observed in our *in vivo* model, we further investigated the relationship between CCL2 and JNK. In cultured murine melanoma cells, we found that CCL2 was released in the medium suggesting that it is produced by cells and it might act in sustaining survival by autocrine/paracrine mechanisms in the tumor microenvironment. When we treated human melanoma cells with recombinant-CCL2, we observed that the chemokine could trigger JNK activation as phosphorylation of JNK1/2 was found. Under such conditions, we did not observe modulation of IKBα, known to be down-regulated following NF-kB activation, and of AKT, whose phosphorylation remained unchanged after recombinant-CCL2 treatment. Thus, a specific CCL2-driven JNK1/2 activation occurred in our model. Moreover, JNK appeared to sustain growth of melanoma cells exposed to TMZ, because upon pharmacological JNK-inhibition, a synergistic effect could be obtained, similarly to what observed with SAHA, further corroborating that JNK pathway impairment is implicated in inhibition of melanoma cell growth.

In addition to modulation of CCL2 by the combination, we observed down-regulation of key inflammatory mediators (i.e., IL-1α, TNFα, TGFβ and VEGF). HDAC inhibitors have been reported to affect the levels of the angiogenic cytokine VEGF in different tumor types by a down-regulation of gene transcription and activation of proteasome activity [34, 35]. In the *in vivo* model we observed a decrease in VEGF transcript after SAHA. Moreover, HDAC inhibition has been shown to suppress TGFβ−induced epithelial to mesenchymal transition [36]. Here, we found a down-regulation of TGFβ levels in tumors from mice treated with SAHA alone and in combination with TMZ. This could be explained by the regulation of TGFβ-induced genes by HDAC and with the effect on VEGF, a TGFβ−target gene, modulated at the transcript/protein levels.

It was previously shown that HDAC inhibitors (including SAHA) sustain the generation and activation of Tregs and MDSC [37, 38]. Conversely, TMZ was reported to decrease Treg frequencies in melanoma patients when applied at low doses [39]. Tregs cells were previously shown to act in the inhibition of the anti-melanoma immune response [25, 40-46]. Moreover, both MDSC and Tregs are known to express receptors for CCL2, CCL4 and CCL5 [25, 43-47]. Therefore, we focused our attention on these major immunosuppressive compartments in the tumor microenvironment, MDSC and Tregs. Here, we demonstrated that a decrease in levels of chemokines positively correlated with reduction in the frequencies of tumor-infiltrating of CD11b^+^Gr1^+^ immature myeloid cells. Previously, we have intensively investigated the distribution and function of CD11b^+^Gr1^+^ cells in primary tumors and metastases from *ret* transgenic mice. We have found that these cells are highly immunosuppressive as reflected by a strong production of nitric oxide, enhanced expression of arginase-1 and their ability to inhibit T cell proliferation in an *in vitro* assay [48-50]. Although we found a decrease in TGF-β levels, a leading factor for the Treg generation [48], and in concentrations of corresponding Treg-chemoattractive proteins in primary tumors, amounts of tumor-infiltrating Tregs were increased in all regimens. Such differences between distribution of MDSC and Tregs might suggest that the pool of MDSC is renewed in the tumor, whereas Tregs are not attracted but converted from the conventional CD4 T cells. Differences in infiltrate did not appear to depend on tumor size, as specimens were harvested 5 days after 5 day treatment from mice with advanced disease and the tumor size of the different groups was similar.

Our findings showing that the levels of CCL2 are decreased in our *in vivo* model after combined treatment, together with a tendency of MDSC to decrease, suggested that the combination of SAHA and TMZ might be endowed with a direct effect on the tumor and with an indirect effect on the microenvironment, as MDSC are attracted by CCL2 to the tumor site [51].

In conclusion, our results showing that down-regulation of CCL2-driven signals *via* JNK contributes to reduce growth of melanoma cells provide a rationale for the therapeutic advantage of the combination of SAHA and TMZ (Figure 6E). The tumor microenvironment may favor tumor growth inhibition because of down-regulation of tumor-associated chronic inflammatory mediators, in particular CCL2, and reduced MDSC recruitment. Since CCL2 is a key modulator of cell survival and its down-regulation has been related to vemurafenib efficacy [4], our findings can be of high translational value. Thus, therapeutic strategy based on HDAC inhibition may be effective because of interference both with tumor cells and with tumor microenvironment-related features.

## MATERIALS AND METHODS

### Ethics Statement

Investigation has been conducted in accordance with the ethical standards and according to the Declaration of Helsinki and according to national and international guidelines and has been approved by the authors' institutional review board.

### Cell lines

The human melanoma cell lines A375 (ATCC CRL1619), 501Mel and the recently established melanoma cell lines (LM17, LM20, LM36 LM18, LM23) were cultured in RPMI-1640 medium plus 10% FBS [52, 53]. All cell lines were authenticated using the Stem Elite ID System (Promega) between February and August 2013, except than A375 cell which were purchased from ATCC. The murine melanoma cell line designated as Me1482, established from a tumor developed in a mouse F1 hybrid carrying the *ret* transgene (see below), was grown in DMEM medium containing 1 mM sodium piruvate plus 10% FBS. The cell line which displayed a fusiform morphology and expressed TRP2 (Supplementary Figure S5), was characterized using a panel of PCR primers for microsatellites polymorphisms between C57BL/6 and BALB/C inbred strains (Supplementary Table S2).

### Drugs

TMZ, SAHA and the JNK-inhibitor SP600125 (Selleck Chemicals, Aurogene Srl, Rome, Italy) were primarily dissolved in DMSO and diluted in water. For in vivo studies, SAHA was dissolved in DMSO/ethanol/PBS (10:5:85) and administered in a volume of 10 mL/kg. Human CCL2/MCP-1 monoclonal neutralizing antibody [54] (cat. 24822, R&D System, SPACE Import Export srl, Milan, Italy), was reconstituted in sterile PBS at 0.5 mg/ml. Recombinant human MCP-1/CCL2 (Peprotech, tebu-bio SRL, Milan, Italy) was reconstituted in sterile water at 0.02 mg/ml.

### Cellular and biochemical assays

Cell sensitivity to drugs was measured by growth-inhibition assays [55]. Twenty-four h after seeding cells were exposed to drugs for 72 h. After treatments cells were counted with a cell counter. At least 3 independent experiments were performed for each mode of treatment. Twenty-four h after seeding cells were exposed to TMZ, SAHA or their combination for 48 h or longer. After the treatment floating and adherent cells were harvested and processed for apoptosis evaluation by TUNEL (Roche, Mannheim, Germany). MCP-1/CCL2 levels in murine and human melanoma cell lines were measured by ELISA according to manufacturer's protocols (ELISA KIT MCP-1, LiStarFish, Milan, Italy).

### Preparation of tumor lysates

To examine biological response to treatments mice with advanced disease (age = 155-172 days) were used. Mice were treated with vehicle, SAHA, TMZ or their combination for 5 days and they were sacrificed 5 days later. Tumor cells were processed for total protein extraction. Briefly, protein lysates were prepared from frozen tumor samples pulverized by the Mikro-Dismembrator II (B. Brown Biotech International, Melsungen, Germany) in Lysis Buffer 15 (from mouse Phospho-RTK Proteome Profiler, R&D System) added with protease and phosphatase inhibitors. Equal amounts of lysates were separated by SDS-PAGE and then transferred to nitrocellulose filters. Filters were incubated with antibodies to phosho-PDGF receptor (Tyr849 and Tyr857; cat. 3170, Cell Signaling Technology, Danvers, MA), phospho-RET (Tyr1062; cat. sc-20252-R, Santa Cruz Biotechnology, Dallas, TX), JNK2 and phospho-JNK1/2 (Thr183/Tyr185; cat. MAB 1846 and AF 1205, R&D System).

### Western blot analysis

Samples were fractionated by SDS-PAGE and blotted on nitrocellulose membranes. Blots were pre-blocked in PBS containing 5% (w/v) dried non-fat milk and then incubated overnight at 4°C with antibodies to JNK2 and phospho-JNK1/2 (R&D System), AKT2 and phospho-AKT2 (cat. AF 23151 and MAB 887; R&D System), NF-kB (cat. sc-109, Santa Cruz Biotechnology) and IKB-a (cat. ab 7545, Abcam Limited, Cambridge, UK). Anti-tubulin, or anti-vinculin antibodies (cat. T9026, and cat. V9131, SIGMA Chemicals Co.) were used as control for the protein loading. Antibody binding to blots was detected by chemiluminescence (Amersham Pharmacia Biotech., Cologno Monzese, Italy). For Proteome Profiler Antibody Arrays, cell lysates were prepared as described by the manufacturer (R&D System). Labscan imaging technology and software (GE Healthcare, Wauwatosa, Wisconsin) was used for Western immunoblot anlysis visualization. Band intensities were quantified by ImageJ software by pixel-integrated intensity.

### Antitumor activity studies

C57BL/6 mice carrying the human *ret* trangene under the control of mouse metallothionein-I promoter, kindly provided by Dr. T. Dragani, were obtained as described previously [56]. Mice were maintained in rooms with controlled temperature and humidity and had free access to food and water. Experiments were approved by the Ethics Committee for Animal Experimentation of the Istituto Nazionale dei Tumori of Milan, according to international guidelines. The general performance of mice was monitored biweekly and spontaneous tumor development was assessed macroscopically; for ethical reasons, animals were sacrificed prior to appearance of signs of suffering. To carry out antitumor activity studies N3/RET mice were crossed to C57BL/6 female mice (Charles River, Calco, Italy) and the resulting F1 mice were genotyped following DNA extraction from a tail fragment to select for the presence of the *ret* transgene. *Ret* transgenic mice developed melanoma starting from 6 weeks. Six-seven week old 48 F1 mice carrying the *ret* transgene showing no signs of macroscopic tumors were used for antitumor activity studies and were assigned to four groups according to a randomization list. The mice were treated (qdx5/wx3w) with a) 50 mg/kg TMZ or b) 100 mg/kg SAHA or c) their combination or d) vehicle. All controls displayed macroscopic evidence of disease (i.e., subcutaneous nodules, exophthalmus) within 44 days from the experiment start.

### Gene expression analyses

TaqMan® Arrays (Mouse Immune Panel, Life Technologies) and qReal-Time PCR assay are described in Supplementary Methods.

### LDH analysis

This method is reported in the Supplementary Methods section.

### Flow cytometry

Tumors, bone marrow, spleen and lymph nodes were harvested into cold PBS, cut into pieces and mechanically disaggregated. Single cell suspensions were obtained by mechanical disaggregation of the specimens that after harvesting from mice were immediately transferred into PBS, cut into pieces and the obtained cell suspensions filtered through 50 μm cell strainers (BD Biosciences). After counting, cells were viably frozen in 10% DMSO in FBS. Thawed cells were washed in PBS and treated with Fc-blocking solution (rat anti-mouse CD16/CD32 antibody, BD Biosciences) followed by the incubation with directly-conjugated monoclonal antibodies (mAbs): CD11b-PE (cat. 553311), Gr1-PE-Cy7 (cat. 552985) and CD45.2-PerCP-Cy5.5 (cat. 552950) from BD Biosciences. To stain FoxP3 cells were fixed and permeabilized using FoxP3 staining set (eBioscience) prior to the addition of the Foxp3-PE mAbs (cat.72-5775-40). Acquisition was performed by multi-color flow cytometry using a FACSCantoII with FACSDiva software (BD Biosciences), with dead cells exclusion based on scatter profile. FlowJo software (Tree Star, Ashland, OR) was used to analyze at least 100,000 events per sample.

### Bio-Plex assay

Snap frozen primary tumor and LN samples were mechanically disrupted and treated by lysis solution (Bio-Rad). Protein concentration in lysates was determined using Bradford assay (Bio-Rad). After sonication, samples were centrifuged at 4,500 g for 6 min at 4 °C. Protein concentration in the supernatant was determined using Bradford assay and adjusted to 1000 μg/ml using serum diluent (both Bio-Rad). Amounts of inflammatory factors in tissue lysates (obtained from tissues from mice treated for 5 days and sacrificed 5 days later) were measured by multiplex technology (Bio-Rad).Transforming growth factor (TGF)-β1 was measured using a single plex kit (Millipore) according to manufacturer's protocol.

### Statistical analysis

The effect of the drug combination in cellular studies was evaluated using the Chou and Talalay method [57] in which a combination index (CI) lower than 1 indicates synergism (Calcusyn software, Biosoft, Cambridge, UK). A detailed description of statistical methods is provided in the Supplementary Methods section.

## SUPPLEMENTARY MATERIAL FIGURES AND TABLES


