# Supplementary material for: Histone deacetylase inhibitor-temozolomide co-treatment inhibits melanoma growth through suppression of Chemokine (C-C motif) ligand 2-driven signals
Source: Oncotarget. 2014 Jun 6;5(12):4516–28. doi: 10.18632/oncotarget.2065 (PMC4147342; doi:10.18632/oncotarget.2065)
Supplement: Supplementary file 1 [file oncotarget-05-4516-s001.pdf]

Histone deacetylase inhibitor-temozolomide co-treatment inhibits melanoma growth through suppression of Chemokine (C-C motif) ligand 2-driven signals

Supplementary Material

Gene expression analyses. Tumor samples were lysed using Tissue Lyser and RNA was extracted with the RNeasy Mini Kit (Qiagen, Hilden, Germany), and quantified using the Nanodrop 2000c spectrophotomer and quality-assessed by denaturing gel electrophoresis. cDNA synthesis was performed with the High-Capacity cDNA Reverse Transcription Kit (Life Technologies, Carlsbad, California) employing 2.5 U/μl of MultiScribe Reverse Transcriptase and 1 μg RNA. The cards (TaqMan® Array Mouse Immune Panel, Life Technologies) were placed in the Micro Fluidic Card Sample Block of an ABI Prism 7900 HT FAST Real Time (Applied Biosystems). The thermal cycling conditions were 2 min at 50°C and 10 min at 95°C, followed by 40 cycles of 30 sec at 97 °C and 1 min at 60 °C. The assay for each gene of the panel was carried out in duplicate, due to the design of this specific panel. The SDS software (Life Technologies) was employed to calculate the threshold cycle (Ct) values. In particular, the baseline was automatically set, while the threshold was manually set to 0.2. The ExpressionSuite software (Life Technologies) was then used to calculate the relative quantification (RQ) values. Relative quantification (delta delta Ct method) was carried out after normalization with respect to a pool of housekeeping genes. The average value obtained from untreated mice was used as calibrator.

LDH analysis. Thirty six mice obtained from F1 of N3/RET mice crossed to C57BL/6 female mice were used for analysis of LDH values. After genotyping, 22 healthy mice (6 females and 16 males) not carrying the transgene were enrolled in the control group, whereas 14 transgenic mice (7 females and 7 males) were enrolled in the case group. Mice were bled from retro-orbital sinus under anaesthesia. Plasmatic lactate dehydrogenase (LDH) enzymatic activity was measured in

heparinized plasma by a routine clinical chemistry analyzer following the manufactures instruction (Architect, Abbott).

Statistical analysis. The effect of the drug combination in cellular studies was evaluated using the Chou and Talalay method in which a combination index (CI) lower than 1 indicates synergism (Calcusyn software, Biosoft, Cambridge, UK). The distribution of each of the considered continuous variables (CCLs, IL1-□, TNF-□, TGF-□, VEGF, percent of leukocytes according to Tumor MDSC and Tumor infiltrating Treg and percent of all cells according to Tumor infiltrating leukocytes) were compared between experimental groups by using the Kruskal-Wallis test (KWT) (1). Bonferroni's correction was used to adjust for multiple comparisons (2). Disease-free survival (DFS) was calculated as the time from experiment start to the first evidence of disease (subcutaneous nodules, exophthalmus). The pattern of DFS was estimated using the Kaplan–Meier method, and the survival curves were compared using log-rank test (3). The comparison of the distribution of LDH values between transgenic mice and healthy mice was carried out by using the Kolmogorov-Smirnov test (4). The relationship between LDH and the disease status was investigated by resorting to a logistic regression model both unadjusted and adjusted by age and sex by using an approach previously described (5). All statistical analyses were carried out with the SAS software (Version 9.2.; SAS Institute, Inc., Cary, NC) by adopting a significance level of $\alpha = 0.05$.

1. Hollander M, Wolfe DA. Nonparametric Statistical Methods. Second Edition, New York: John Wiley & Sons, 1999.
2. Brown BW, Russell K. Methods of correcting for multiple testing: operating characteristics. Stat Med. 1997; 16:2511-2528.

3. Kaplan EL, Meier P. Nonparametric estimation from incomplete observations. J Amer Statis Assn 1958; 53:457–481.
4. Hollander M, Wolfe DA. Nonparametric Statistical Methods. Second Edition, New York: John Wiley & Sons, 1999.
5. Orlandi R, De Bortoli M, Ciniselli CM, Vaghi E, Caccia D, Garrisi V, et al. Hepcidin and ferritin blood level as noninvasive tools for predicting breast cancer. Ann Oncol. 2014; 25:352-7.

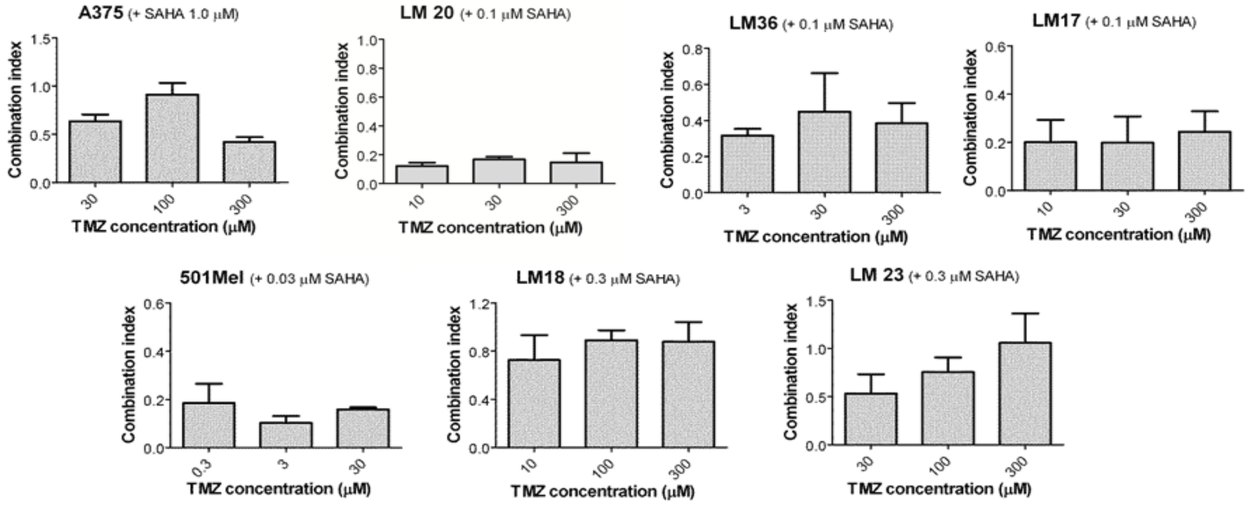

Supplementary Figure S1. Analysis of the drug interaction in melanoma cell lines.
Cell sensitivity to temozolomide (TMZ), SAHA or to their combination was assessed by growth inhibition assays. Cells were exposed for 72 h to each drug alone or to the drug combination. Histograms of the mean of Combination Index values of at least 3 independent experiments for the different cell lines are shown.

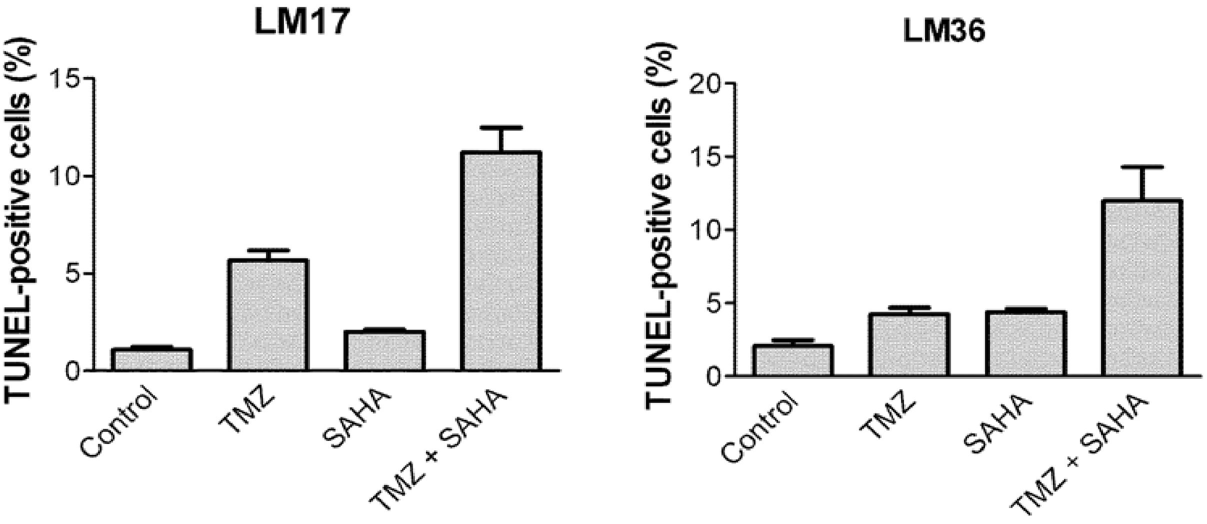

Supplementary Figure S2. Analysis of apoptosis induction in melanoma cell lines. Quantitative analysis of apoptosis induction by TUNEL assay in LM17 and LM36. LM17 were exposed to 300 µM TMZ, 1 µM SAHA or to their combination and processed 72 h later. LM36 were exposed to 30 µM TMZ, 0.1 µM SAHA or to their combination and processed 144 h later.

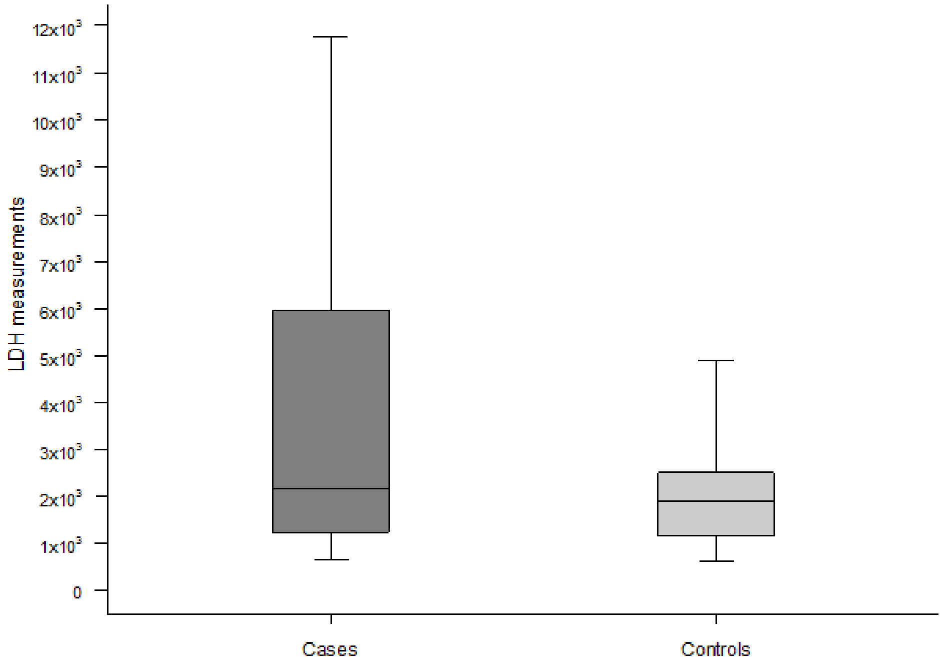

Supplementary Figure S3. **Boxplot of LDH measurements**
Box plots reflecting the distribution of LDH in transgenic mice (cases) and in healthy mice (controls). Each box indicates the 25th and 75th centiles. The horizontal line inside the box indicates the median, and the whiskers indicate the extreme measured values.

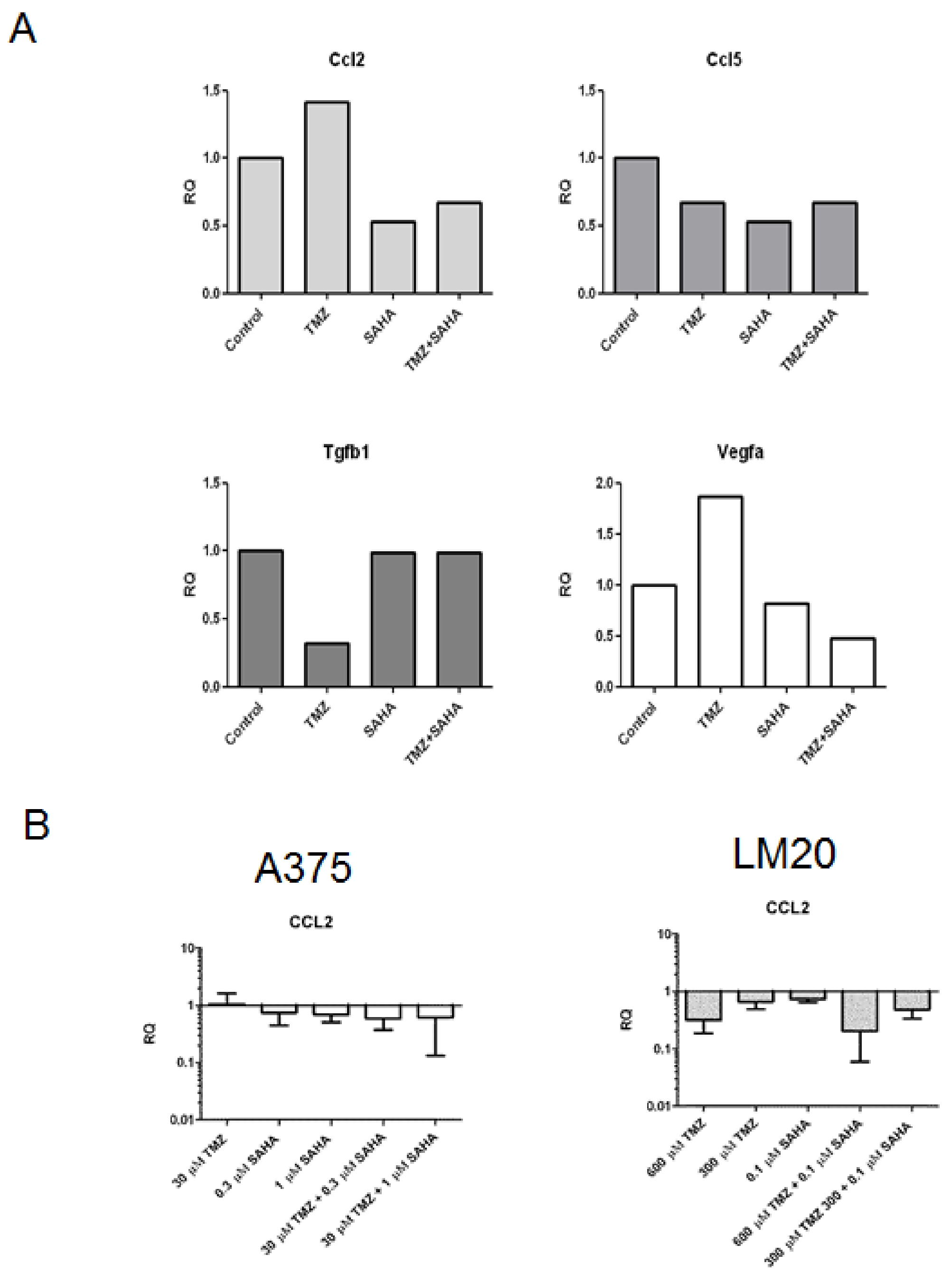

Supplementary Figure S4. Modulation of mRNA levels of selected genes by temozolomide, SAHA or their combination in murine melanoma specimens and melanoma cell lines. (A) Gene expression analysis of different cytokines in murine tumor specimens was carried out with TaqMan® Array Mouse Immune Panel. Tumors were harvested 5 days after 5 days of simultaneous treatment and processed for RNA extraction. (B) The mRNA level of CCL2 gene in A375 and LM20 cells was analyzed by qReal-Time PCR. RQ values (treated vs untreated cells) are reported.

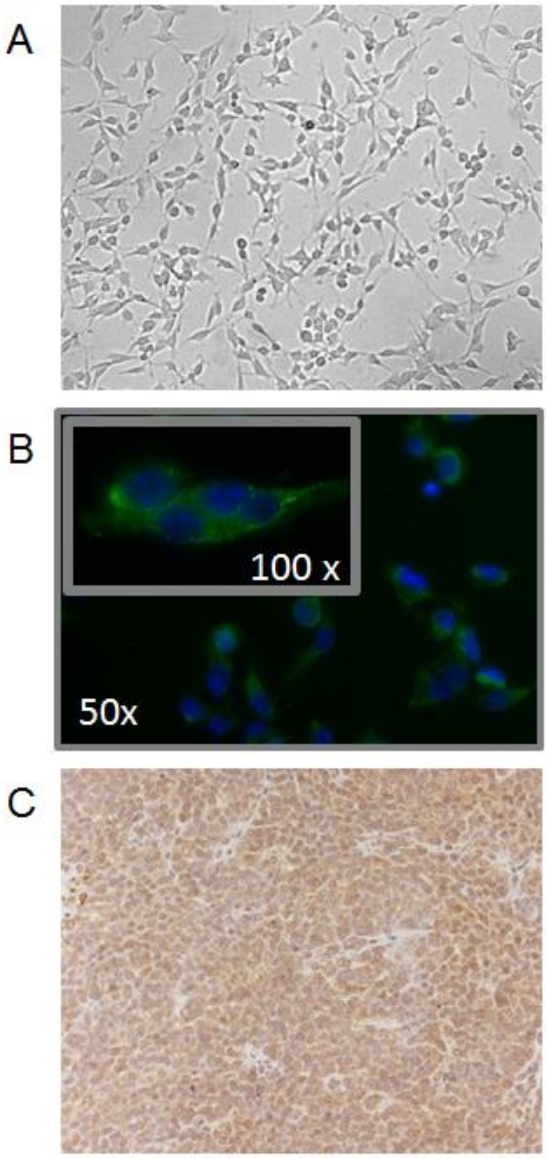

Supplementary Figure S5. Characterization of murine Me1482 melanoma cells

(A) Morphology of the Me1482 cell line. (B) Immunofluorescence staining for TRP2 (Abcam, Cambridge, UK). (C) IHC analysis of TRP2 in a tumor derived from *ret* mice. The Leica DMRB microscope was used, together with the IAS2000 acquisition software (Delta Sistemi, sas, Rome).

Supplementary Table S1. Descriptive statistics for LDH measurement obtained in plasma from transgenic *Ret* mice and controls[a]

	n	min	25th centile	median	75th centile	max	IQR	p-value [b]
Controls	22	606	1158	1887	2502	4902	1344	0.172
Cases	11*	660	1158	2160	7122	11772	5964	

*For three cases the LDH measurements was not available;

IQR, Interquartile range= (75th centile -25th centile)

[a]Hybrid mice obtained crossing C57BL/6 female mice by N3/RET mice were used for analysis of LDH values. The median age of the *ret* transgenic mice was 161 days in both the cases (range: 154-166 days) and controls (range: 154-179). After genotyping, healthy mice not carrying the transgene were enrolled in the control group, whereas mice carrying the transgene were enrolled in the case group.

[b] Kolmogorov-Smirnov Test.

Supplementary Table S2 – Genetic characterization of the Me1482 cell line [a]

**Marker**	**Map**	**Primer sequence**	**Me1482 genotype**
**D1Mit84**	chr1:91909897-91910147	**TGTCTCCCCAAAGTAGCAGG**	B/C
**D1Mit84**		**GTGATGCAGGAGTTTCTGCA**	
**D1Mit365**	chr 1:92129184-92129282	**ATCACCTGCAATAGTACCCCC**	B/C
**D1Mit365**		**TTAATCAGTCATCATAGGCTTTTCC**	
**D2Mit328**	chr2:71963824-	**CTTTCAATGTTCCGGCATG**	B/B

	71964058		
**D2Mit328**		**AAGACTTGCTTTCATTAGACCACA**	
**Kras 37bpRepeat**	chr 6:	**GATGGCATCTTGGACCTTACTC**	B/C
**Kras 37bpRepeat**	chr 6:	**AGTCTGCGTGCGCTTGTAA**	
**D6Mit372**	chr6:148450482-148450593	**TTAATACACTTAGGTGTGGCTCTCC**	B/C
**D6Mit372**		**GAGAGGCATATAGAAAAGGATAATGC**	
**D11Mit54**	chr11:96285639-96285776	**AGGCTGGTGGCTAGTGTCC**	B/B
**D11Mit54**	chr11:	**AAGTCTTGCGCTGCATCTTT**	
**D18Mit210**	chr18:75843369-75843487	**TGGGCAGAAGTATAACTAAATCCA**	B/C
**D18Mit210r**		**TTCAAACCGTATGCCTTTCC**	

[a]Genetic characterization of polymorphisms was carried out by PCR. B refers to C57BL/6 background, C to BALB/ c background. Map as from Ensembl release 73 - September 2013
